# Psychological, cognitive factors and contextual influences in pain and pain-related suffering as revealed by a combined qualitative and quantitative assessment approach

**DOI:** 10.1371/journal.pone.0199814

**Published:** 2018-07-31

**Authors:** Smadar Bustan, Ana Maria Gonzalez-Roldan, Christoph Schommer, Sandra Kamping, Martin Löffler, Michael Brunner, Herta Flor, Fernand Anton

**Affiliations:** 1 INSERM U-987, CHU « Pathophysiology and Clinical Pharmacology of Pain» Hospital Ambroise Paré, Boulogne-Billancourt, France; 2 Department of Cognitive and Clinical Neuroscience, Central Institute of Mental Health, Medical Faculty Mannheim, University of Heidelberg, Mannheim, Germany; 3 Institute for Health and Behavior, FLSHASE/INSIDE, University of Luxembourg, Esch-sur-Alzette, Luxembourg; 4 Research Institute on Health Sciences (IUNICS), University of Balearic Islands, Palma de Mallorca, Spain; 5 ILIAS Laboratory, Dept. of Computer Science and Communication, FSTC, University of Luxembourg, Esch-sur-Alzette, Luxembourg; University of Technology Sydney, AUSTRALIA

## Abstract

Previous psychophysiological research suggests that pain measurement needs to go beyond the assessment of Pain Intensity and Unpleasantness by adding the evaluation of Pain-Related Suffering. Based on this three-dimensional approach, we attempted to elucidate who is more likely to suffer by identifying reasons that may lead individuals to report Pain and Pain-Related Suffering more than others. A sample of 24 healthy participants (age range 18–33) underwent four different sessions involving the evaluation of experimentally induced phasic and tonic pain. We applied two decision tree models to identify variables (selected from psychological questionnaires regarding pain and descriptors from post-session interviews) that provided a qualitative characterization of the degrees of Pain Intensity, Unpleasantness and Suffering and assessed the respective impact of contextual influences. The overall classification accuracy of the decision trees was 75% for Intensity, 77% for Unpleasantness and 78% for Pain-Related Suffering. The reporting of suffering was predominantly associated with fear of pain and active cognitive coping strategies, pain intensity with bodily competence conveying strength and resistance and unpleasantness with the degree of fear of pain and catastrophizing. These results indicate that the appraisal of the three pain dimensions was largely determined by stable psychological constructs. They also suggest that individuals manifesting higher active coping strategies may suffer less despite enhanced pain and those who fear pain may suffer even under low pain. The second decision tree model revealed that suffering did not depend on pain alone, but that the complex rating-related decision making can be shifted by situational factors (context, emotional and cognitive). The impact of coping and fear of pain on individual Pain-Related Suffering may highlight the importance of improving cognitive coping strategies in clinical settings.

## Introduction

The assessment of pain typically focuses on the degree of pain intensity (I) and unpleasantness (U)[1]. Our previous studies with healthy volunteers suggest that an extension to a three-dimensional measurement comprising pain-related suffering (PS) is desirable [2,3] and better captures the clinical situation [4–6]. Particularly, we assume that difficulties in clinical pain evaluation such as frequently observed extremely high pain ratings could be related to not explicitly documented levels of suffering that often remain confounded with pain intensity and unpleasantness [7]. Suffering as an important dimension of pain was integrated in many theoretical models of pain [4, 8–14] however, its inclusion in experimental and clinical studies of pain has been scarce partly due to the lack of viable assessment methods. A combined quantitative and qualitative evaluation of suffering in an experimental context mirroring clinical pain conditions may better depict the experience of pain as a whole.

Predominantly, the suffering aspect is estimated to encompass emotional and cognitive qualities of pain which unpleasantness does not fully embody. Melzack and Wall [15] emphasized that “what is missing in the word ‘unpleasant’ is the misery, anguish, desperation, and urgency that are part of some pain experiences”. Suffering has since been conceptualized either as an enhanced form of pain unpleasantness or as an additional element combining several negative emotions [14, 13, 16] labelled “pain-related extended emotions” [11, 17–19], but not as an independent pain component in its *full* scope.

In our previous investigations assessing suffering in an experimental context, participants received either series of tonic and phasic noxious mechanical stimuli [2] or of tonic thermal and phasic electric stimuli [3]. Unpleasantness and suffering ratings were repeatedly shown to be higher in response to tonic as compared to phasic pain stimuli which both relied on deep pressure stimulation aimed at mirroring clinical pain as closely as possible. Visual analogue scales were used to rate intensity, unpleasantness and suffering for low, moderate and high pain conditions. In this context, we deliberately did not propose prefixed definitions of pain intensity, unpleasantness or suffering in order to allow for the diverse properties of pain and suffering to emerge spontaneously according to individual conceptions and on-site experiences in the laboratory. Principal component analyses confirmed that even though the intensity ratings explained most of the variance, the suffering ratings constituted an integral component of pain processing that was independent from intensity and unpleasantness, thus constituting a separate third component of the pain experience. The emergence of suffering in young healthy financially compensated volunteers, despite ethical guidelines-related restrictions of experimentally induced pain, supported the idea that suffering is a prominent characteristic of pain per se.

Based on the described three-dimensional approach, the objective of the present study was to identify factors, which, besides the respective pain conditions, may enhance the reporting of pain and pain-related suffering. Given the established impact of psychological variables such as cognitive evaluation and emotional response, involving fear of pain, catastrophizing or coping-strategies on pain processing [20–25], these stable variables were also expected to have robust effects on suffering that has scarcely been investigated in this respect. However, since people differ in their understanding and judgment of pain and suffering, personal meanings they assign to these concepts [26,27] and contextual variables modulating their perception may also have an important influence.

To depict the most prominent factors, we first undertook to determine the nature of each pain component by recording the meaning participants attributed to “Intensity”, “Unpleasantness” and “Suffering” via descriptors collected from 96 interviews conducted after the phasic and tonic mechanical pain stimulation protocols described in Bustan et al. [2]. Relating numerical measures to descriptive interpretations allowed to ascertain the conceptual and semantic validation of each pain dimension.

We secondly sought to provide a classification of the various factors according to their weight on the pain-suffering ratings by using the decision tree technique [28], a common tool in medicine for the assessment of decision-making processes of patients [29]. This classification technique has the benefit of associating quantitative and qualitative assessment scales and is thus a highly adequate data mining approach for clarifying the predictive value of predominant psychological constructs and situational variables for the ratings of pain intensity, unpleasantness, and suffering. Accordingly, two classification models were automatically developed [30,31] by combining categorical variables of pain-suffering meanings from the qualitative interview descriptors as well as the psychological variables from the questionnaires data with numerical variables of high versus low I-U-PS psychophysical scores (target values). Whereas the resulting first concise “pruned tree” model selected the most predisposing factors for each pain dimension and condition, the goal of the second exploratory “unpruned tree” model was to elucidate whether situational factors (relating to environment, pain duration and induction method, mood and emotive-cognitive states) may modulate the rating decision process. We expected shifts in the course of the rating decisions that were related to the impact of context and of attention directed to the actual pain experience. We also expected a concomitant reduction of the weight of the identified psychological factors. The overall goal of this study was to identify factors affecting the reporting of pain and related suffering and allowing us to identify participants that are more prone to suffer.

## Methods

### Participants and design

This study is based on data sets additionally recorded from the participant sample described in a previous paper [2]. Twenty-four healthy, right-handed students (12 men, 12 women), aged 18–33 years (mean = 22.50, standard deviation (SD) = 3.61), participated in the study and received financial compensation. Thirty-four volunteers fulfilled the inclusion criteria and participated in the study, however, 10 had to be excluded either because they did not complete the four sessions of the experiment having decided to drop out (N = 4) or due to technical problems with the psychophysiological recordings (N = 6). The participants were assessed for mental disorder based on the Structured Clinical Interview for DSM-IV (SCID-I) screening module [32]. No subject had a current or prior history of acute or chronic pain, chronic somatic diseases, lifetime or current mental disorder or pregnancy and no one used analgesic medication.

The study was conducted in accordance with the declaration of Helsinki and the ethical guidelines of the International Association for the Study of Pain [33] and was approved by the National Research Ethics Committee of Luxembourg (ref. 1102–59). The participants were given a detailed explanation of the experimental procedure and signed written informed consent. They were informed that they could decide to terminate the experiment at any time (chosen by 4/34 subjects).

### Questionnaires

All participants were of Western European origin and completed two sets of questionnaires (either in the German version for 11 Luxembourgish and German mother tongues or in the English version for the other 13 participants who were all fluent in English or native speakers (6)). One week before the experiment, the participants completed online psychological measures which included: 1) The Fear of Pain Questionnaire (FPQ-III [34,35]) measures the fear of pain according to the severity of the expected form of pain. It uses three subscale scores for assessing fear of minor pain (e.g. biting your tongue), severe pain (e.g. breaking a leg) and medical pain (e.g. to get a penicillin shot) as well as their total score. The scoring range for each subscale is 10 to 50 and for the total score is 30 to 150 with higher scores indicating more fear. 2) The Pain-Related Self-statements Scale (PRSS [36]; FSS [37]) assessing the patients' cognitive coping with pain and consisting of two subscales: “catastrophic thoughts (catastrophizing)” and “active controlling thoughts (coping)” to evaluate situation-specific cognitions that either promote or hinder attempts to cope with pain (scoring range 1 to 5). 3) Considering that suffering may be reflected in how people relate to their body and the inter-individual differences in how people access and interpret the private and public aspects of their selves but also given the literature on the well-established relationship between pain and body consciousness reporting, it can be predicted that individuals prone to high levels of attentional self-focus and negative affect will declare more pain [38,39]. In order to assess individual differences in attention to internal physical sensations and their relation to pain-related suffering, the respective differences were divided into private and public-social self-consciousness as measured by the Self-Consciousness Inventory by Fenigstein, Scheier and Buss [40]. To apply this private-public distinction to the body, the Body-Consciousness Questionnaire (BCQ [41,42]) was used. It divides body-consciousness into the three scales: Private Body-Consciousness (tendency to focus on internal body sensations), public body-consciousness (consciousness of the body as perceived by an observer) and body competence (an individual's sense of body effectiveness and strength, e.g. “I am capable of moving quickly” or “for my size, I am pretty strong”) and measures the degree to which a person concentrates on inner bodily experiences and tensions or on his outer social and aesthetical appearance. The scoring range for each subscale is: Private 0–20, Public 0–24 and Body Competence 0–16 respectively. Since body competence is a significant third factor revealed by this questionnaire, we were able to measure the extent of confidence a person has about his/her own body. 4) The Edinburgh Handedness Inventory [43] assesses the dominance of a person’s right or left hand. The mean scores of this set of questionnaires can be seen in the S1 Table of the supplementary material.

The second set of questionnaires was completed twice during each of the four experimental sessions (before the baseline recording and after the threshold measurement) and evaluated the mood state during the experiment by asking the participants to rate valence, arousal, and dominance using the Self-Assessment Manikin (SAM)[44]. The participants also completed the Positive and Negative Affect Schedule (PANAS)[45,46]) with the two factors labelled “Positive Affect” and “Negative Affect” testifying reliably as the dominant dimensions of emotional experience. The two factors are subject to variations depending on situational context, time, culture and languages. Participants additionally used a 5-point Likert scale (from “not at all” to “extremely”) to answer the following two questions: “How worried are you that something serious might happen?” and “How afraid are you of the following pain induction?”. The mean scores of this set of questionnaires can be seen in the S2 Table of the supplementary material.

### Pain induction procedures

Briefly, mechanically induced pain was measured in response to both phasic (pneumatically driven Impact Stimulator, custom built by Labortechnik Franken, Germany with blunt plastic projectiles of mass 307 mg and diameter 12 mm accelerated through a guiding plexiglass tube and applied to the dorsal side of the middle phalanx of the left hand [47,48] and tonic stimuli (Interdigital Web Pinching device consisting of a pneumatically controlled plastic forceps with rounded tips, of diameter 5 mm; modified custom built version of the device used by Forster et al. [49]. The stimuli consisted in the application of pinch pressure to the inter-digital web between the third and fourth digit of the right hand [50] and were vertically applied to prevent any physical harm to the skin. In a subset of experimental protocols, acoustic startle probes were applied concomitantly with the noxious mechanical stimuli in order to probe emotional load. For this purpose, bursts of white noise (105 dB, 50 ms duration, instantaneous rise time, binaural stimulation) were presented via headphones (PD81, Holmberg, Germany).

Each participant completed four sessions (lasting approximately 50 minutes each). In condition 1, startle reflexes were induced during the administration of tonic noxious stimulation. In condition 2, startle reflexes were evoked during the application of trains of phasic stimulation. In conditions 3 and 4, tonic and phasic pain stimuli, respectively, were presented without concomitant induction of startle reflexes. The experiments were performed on two days separated by an interval of one week. On each day, two consecutive sessions were conducted with a 30 minute break in between providing for a standard snack. The sessions were performed on the same time of day (morning or afternoon). The order of the sessions was fully counterbalanced across subjects. The participants rated I, U and PS in response to the noxious stimuli using visual analogue scales (VAS) ranging from no Pain, Unpleasantness or Suffering to extreme Pain, Unpleasantness or Suffering. To make sure that the participants based the pain evaluation on their own pain-related suffering concepts and representations, no previous definitions were given for the respective parameters.

For both mechanical stimulation methods, individual pain thresholds and pain tolerance levels were assessed in four ascending series using the method of limits [51]. They were then computed as the mean of the three last series. Pain threshold was determined as the minimum stimulation intensity required for the induction of a painful sensation and pain tolerance as the maximum intensity the participants were able to endure. The phasic thresholds were determined by presenting trains of 10 stimuli from a baseline impulse of 1.1 gm/s that were increased at a rate of 0.1 gm/s following each 10-s rest interval (cut-off limit was 2.7 gm/s). For tonic threshold measurements, 10-s-lasting stimuli were presented and intensities were increased at a rate of 1 N from a baseline pressure of 6.5 N (to prevent tissue damage, the cut-off limit was set to 16 N). The stimulation periods were separated by breaks of 10 s. For each stimulation method, supra-threshold noxious stimulation levels were determined by calculating the pain sensitivity range (pain tolerance–pain threshold) and then applying three stimulus intensities defined as Low = pain threshold + 40% pain sensitivity range, Mild = pain threshold + 60% pain sensitivity range and High = pain threshold + 80% pain sensitivity range. In addition, two stimulus durations were used. For the tonic stimuli, short stimuli lasted 150 s and long ones 210 s. For the phasic stimuli, short stimulation was set as trains of 10 mechanical impacts, and long stimulation as trains of 30 mechanical impacts.

### Interviews

#### Data collection

After each of the four sessions, identical semi-structured interviews aimed at obtaining qualitative descriptions of the participants’ pain Intensity, Unpleasantness and Suffering were conducted in English. The interviews were identical each time in order to examine whether the participants qualified pain and suffering similarly following the repeated pain measurement with or without the acoustic startle probes. They lasted 20 min in average and were audiotaped, transcribed and analyzed. All participants were Western European students, 6 were English native speakers and all were selected to participate in the study with the requirement of having excellent proficiency in English. The interview comprised six questions devoted to the individual concepts and lived experience of pain and suffering, presented in a randomized order across participants:

1. Can you describe in your own words your experience of pain intensity?
2. Can you describe in your own words your experience of Pain Unpleasantness?
3. Can you describe in your own words your experience of pain-related suffering, if you felt that you were suffering?
4. If answer is affirmative: could you say what type of suffering it was?
5. Can you differentiate between pain and suffering in your experience during the experiment?

(ONLY ASKED AFTER SECOND STIMULATION METHOD) Comparing the two methods, can you differentiate between your suffering and your pain experience?

#### Data analysis

The software package NVivo (qualitative data analysis program, Version 1.3, 2000) was used for processing the interviews. Its coding system allowed to sort the sentences according to their content similarity and frequent repetition in a way that assured standardization [52]. For a thematic content analysis [53–55], the data treatment proceeded as follows. First, in order to avoid any prior influences or preconceptions [56], an analyst blind to theories about pain and pain-elated suffering, who did not know the experimental questions addressed repeatedly, read through each interview and systematically grouped similar ideas under a common descriptor, formulated in adherence to the interviews’ original quotes. At the end of this initial process, 97 descriptors were identified. In a second step, a group of 3 experts (two experts on pain and one on suffering) went through the descriptors (items) and the verbatim text (original quotes from the interviews) to verify the coding. This procedure was first conducted by each expert separately, and then discussed together by all three experts in a round table discussion with revisions conducted based on majority consensus. Items specifically related to the experimental methods or composed of sentences with unclear meaning were rejected. In the third stage, content validation [57,58] was performed individually with 6 articulate persons who had participated in the experiment. They were asked to explain the meaning of the descriptors in order to verify whether or not they were clear and accurate enough for use in an experimental set-up. Based on their input, unclear descriptors were rephrased and ambiguous ones deleted. Moreover, in order to obtain the most pronounced descriptors, we only included those mentioned by at least four participants. Finally, the content validation procedure was again carried out with two independent experts in the field of pain research and a third expert in medical and health sociology. The resulting final list of 41 items most adequately represented the participants’ pain and related suffering experience with descriptors such as: “I cannot take the pain anymore”, “Pain makes me feel angry”, “I do not want to experience what is happening to me”, “I do not know when my pain will end”, “I lack control over the situation”, “The pain was intense and frequent enough to make me suffer” or “Suffering is like a mental strain and exhaustion”.

### Classification trees

#### Interview quantification

The next step was performed in order to attribute quantitative values to the qualitative data of the interviews. For this purpose, two of the experts went over the quotes of each individual and coded them for each of the 41 descriptors as follows: 1 = the participant’s verbal report agreed with the item, 2 = the participant disagreed with the item, 0 = the participant did not mention the item at all.

#### Data mining

In the present descriptive study we used the decision making approach provided by the Classification Trees since it allows the combined analysis of different kinds of variables (numerical, categorical) about the relation between rating and interpreting the experience of pain and related suffering.

The data mining analysis used the decision tree technique that is based on If-Then rules [59–64]. This algorithmic approach has the advantage of employing all the data without hypothesizing about the expected outcome as all hypotheses are considered equally likely a priori. This process of inductive inference starts with the root node of the decision tree and moves down the branches (attribute nodes) until the terminal leaf node is reached, expressing each time a decision. Every branch represents a decision classification based on a binary splitting (2 branches) whereby each hypothesis in the node is either "true" (yes) or "false” (no). For example: IF there is low fear of severe pain–THEN (= True case) there is low pain intensity, else (= False case) there is high pain intensity. Most often, the decision process is built from a set of attributes, for example: IF there is low body competence (BCQs<9.5) and low catastrophic thoughts (PRSSs<1.9) THEN: low pain intensity. In total 58 variables were included for the data processing: 41 descriptors from the interviews, 10 questionnaire subscales from the first set of questionnaires prior to the experiment and 7 questionnaires from the second set completed during the experiment. This pre-selection of only a subset of the total list of variables was due to values of a variable being completely identical, too sparse or missing.

The I, U, PS ratings constituted the target attribute variables defined by the investigators according to the study’s objectives. These target attributes were operationalized by using the median ratings of each scale across the whole experiment (I, U and PS) as cut-off points to assign each subject to the category of "high" or "low" (median values are indicated in Table 1). For both the high and low psychophysical scores, the classification technique allows to determine the associated psychological attributes or experiential factors and hence to depict the meaning participants attributed to “Intensity”, “Unpleasantness” and “Suffering” based on the post-experiment interviews.

**Table 1:** The table displays median values and interquartile range, in parenthesis, of visual analogue scale ratings of intensity, unpleasantness and suffering that are used to classify each subject under the two categories of each target variable (“low” or “high”).

Median values and interquartile range, in parenthesis, Visual Analogue Scale ratings (ranging from “no Pain, Unpleasantness or Suffering” to “extreme Pain, Unpleasantness or Suffering”)			
	Intensity	Unpleasantness	Suffering
**Phasic + Startle**	47.64 (28.32)	49.86 (23.02)	31.59 (27.15)
**Tonic + Startle**	59.55 (28.08)	68.14 (29.02)	49.84 (28.32)
**Phasic Alone**	42.73 (22.25)	43.70 (28.94)	27.48 (26.71)
**Tonic Alone**	64.91 (20.27)	73.22 (36.32)	57.07 (32.04)

Two types of trees’ models were generated by employing the classification and regression tree technique (CART) as originally introduced by the tree induction algorithm of Breiman et al. [30]. In the present study, data mining trees models were constructed using Decision Trees Classification with IBM DB2 Intelligent Miner for Data version 8.1 software [65]. We applied entropy as a measure for splitting and generating the branches inside the decision tree. It was applied to all selected attributes in a 'step by step' manner in order to determine which ones allowed the best differentiation between subjects displaying different target attribute values. The CART process comprised two parts:

First, for each of the four experimental pain conditions, an in-depth mapping of the subjects' data was performed through an unpruned tree model. Each leaf of the tree shared a purity of 100%, implying that it was populated by subjects sharing the same target attribute values. While this unpruned step allows a detailed description of each group, it may suffer from an (inherent) overfitting and an unnecessarily complex tree model. A pruned tree model (presented in model 1) aimed at simplifying the structure of the tree and at enhancing clarity for reasonable interpretation was hence generated by fusing branches. While this commonly performed approach may impinge on the detailed representation of the decision process and hence lead to a reduced classification accuracy [63, 30, 66] of the various factors associated with the pain dimensions and conditions, it assures a more robust model for future data sets. The true positives, true negatives, false positives and false negatives from the training data set constituted the basis for the creation of the model. The error rate was then computed to measure the number of misclassifications.

This classification procedure was then used to determine the reasons for “low” vs. “high” ratings of I, U and PS in each of the 4 session conditions (tonic, tonic+startle, phasic, phasic+startle). Therefore, a total of 12 pruned and of 12 unpruned decision trees were obtained (3 target variables x 4 session conditions). All pruned trees are presented. However, since we were mainly interested in responses related to pain processing, and given the high complexity and descriptive purposes of unpruned trees, only 3 unpruned examples resulting from the pain-only conditions are displayed for the I, U and PS respectively: the I unpruned tree of the phasic condition, the U and PS unpruned trees of the tonic condition.

## Results

In order to identify who is more likely to perceive pain, suffering and for what reasons, our goal with the decision tree construction was to create a model that identifies independent variables that are characteristic for high versus low pain ratings. By combining measures from the I-U-PS ratings as target variables, along with the selected interview descriptors (41) and the data from the psychological questionnaires as possible explanatory variables (reporting first hand immediate impression and the influence of psychological factors on the subjective experience), we obtained 2 classifications with respect to the decision processes underlying the pain evaluations: a pruned model mainly pertaining to psychological constructs on pain and suffering and an unpruned model combining these constructs with situational factors based on subjective variables such as emotional states and first hand impression from the pain attended situation. No significant sex differences were noted for any of the questionnaire data and as previously explained [2], no significant main effects or interactions related to startle or sex in the VAS ratings.

### Pruned trees (predictive model)

The overall classification accuracy across conditions was 75% for I, 77% for U and 78% for PS (see Table 2). In both tonic pain conditions (tonic alone, tonic + startle), low I, U and PS was always better inferred (84.73% mean accuracy) than high I, U and PS (62.49% mean accuracy). In contrast, in both less pronounced phasic pain conditions (with or without startle) high I, U and PS were better predicted (94.4% mean accuracy) than low I, U and PS (61.3% mean accuracy).

**Table 2:** The table displays prediction accuracy values (in %) for high and low intensity, unpleasantness and suffering in the different pain conditions. Prediction accuracy refers to the soundness of the classification model to predict new data correctly.

Prediction Accuracy %												
	Intensity			Unpleasantness			Suffering			Total		
	High	Low	Total	High	Low	Total	High	Low	Total	High	Low	Total
**Phasic + Startle**	91.7	58.3	75.0	91.7	58.3	75.0	91.7	66.7	79.2	91.7	61.1	76.4
**Tonic + Startle**	33.3	100	66.4	58.3	91.7	75.0	50.0	100	75.0	47.2	97.2	72.1
**Phasic Alone**	100	66.7	83.3	100	66.7	83.3	91.7	75.0	83.3	97.2	69.5	83.3
**Tonic Alone**	58.3	91.7	75.0	75.0	75.0	75.0	100	50.0	75.0	77.8	72.2	75.0
**Total**	70.8	79.2	**74.9**	81.3	72.9	**77.1**	83.3	72.9	**78.1**	78.5	75.0	

**Fig 1:**
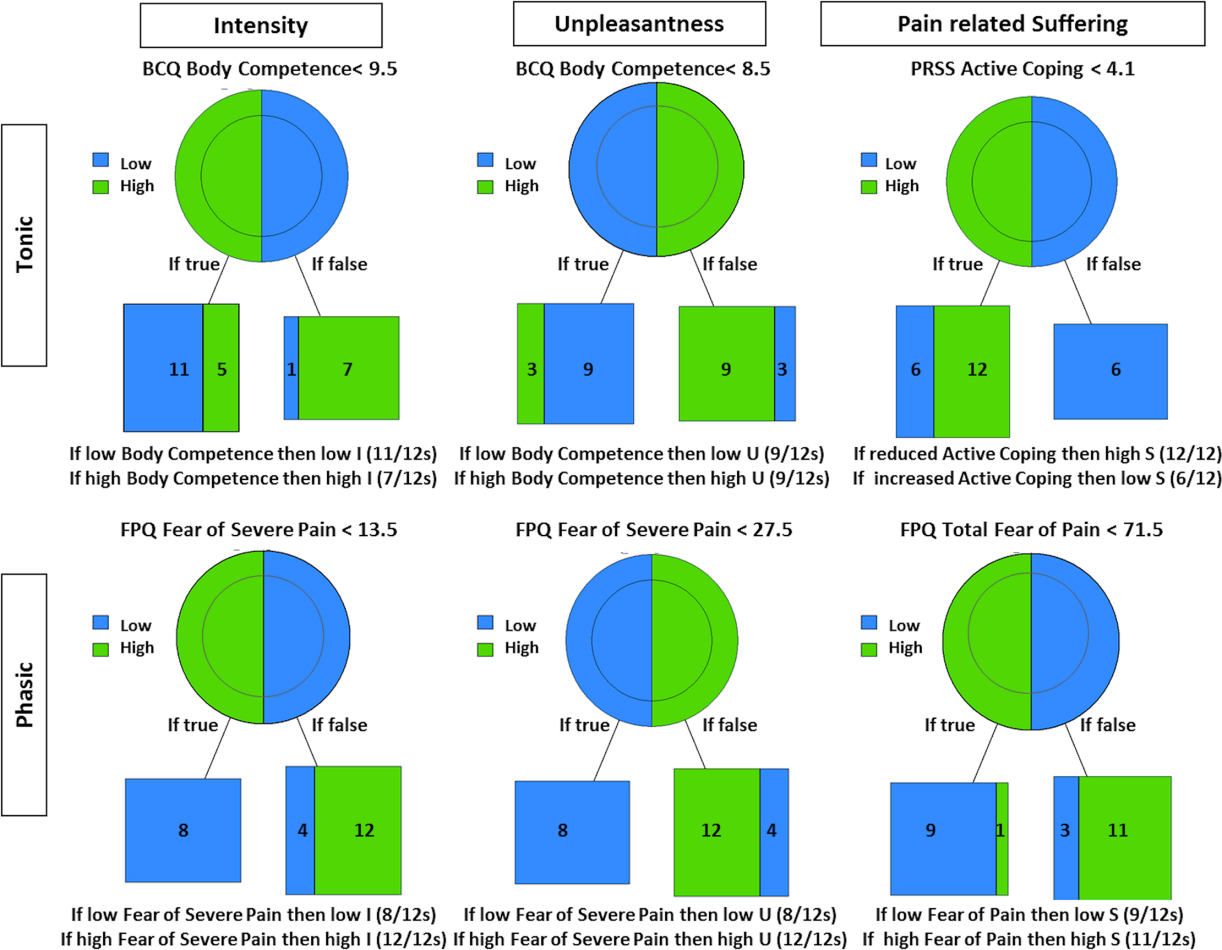
**Pruned decision trees for intensity, unpleasantness and pain-related suffering for the tonic (upper trees) and phasic (bottom trees) conditions. Each tree presents the decision rule (superior line) that predicts the ratings of the subjects as well as the number of subjects predicted under each group (bottom boxes)**. The right leaf of the tree represents the amount of subjects who followed the IF-THEN rule [59–64] in confirming the inference stated (yes), and the left represents the subjects who did not (no).

**Fig 2:**
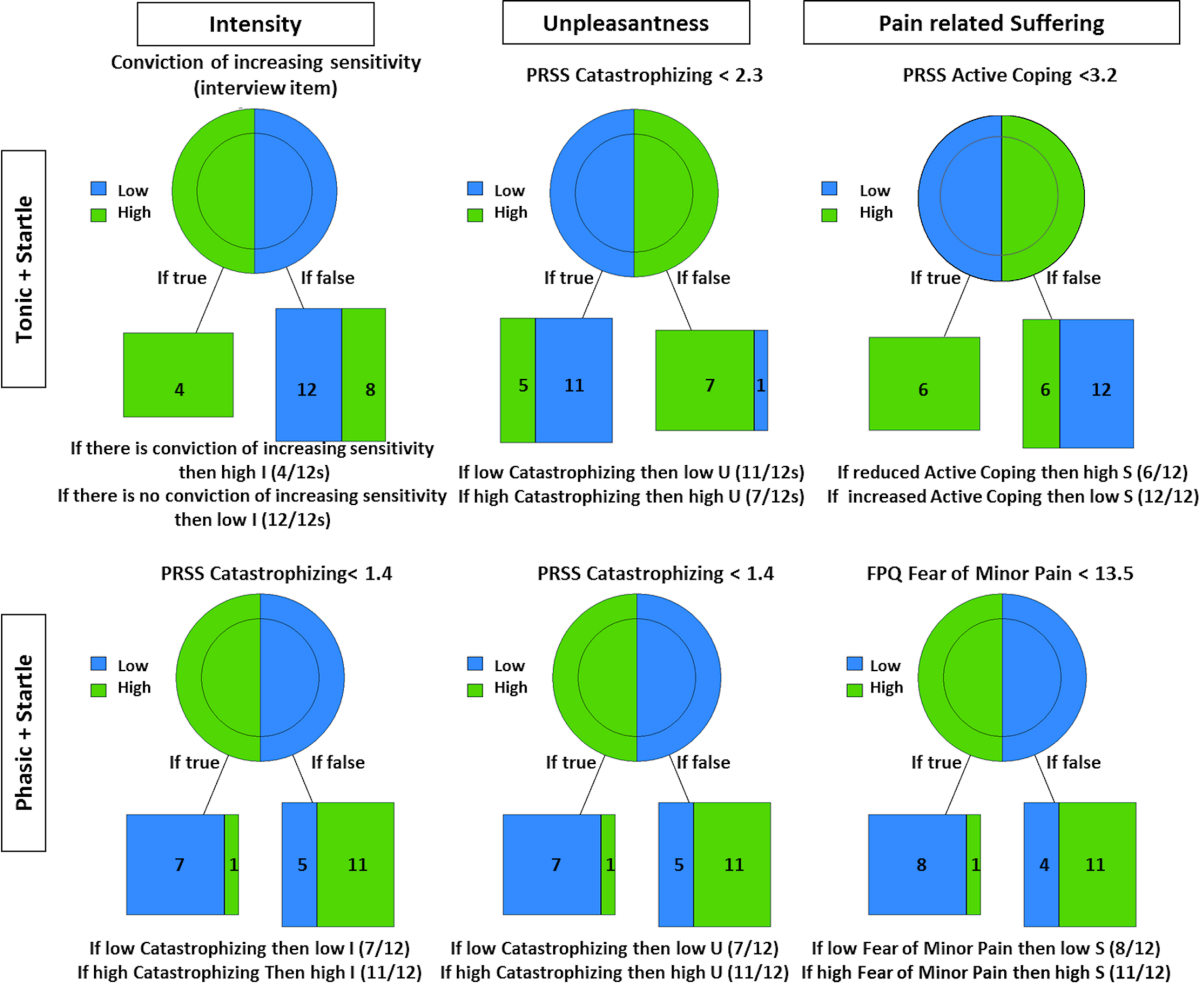
**Pruned trees for intensity, unpleasantness and pain-related suffering for the tonic + startle (upper trees) and phasic + startle (bottom trees) conditions**. Each tree presents the rule (superior line) that predicts the ratings of the subjects as well as the number of subjects predicted under each group (bottom boxes). The right leaf of the tree represents the amount of subjects who followed the rule, and the left leaf the subjects who did not.

For the variables generated in model 1 the psychological constructs and the only situational item from the interview descriptors (“Conviction of increasing sensitivity”) explaining ‘high’ and ‘low’ I, U and PS for each condition and category are shown in Table 3. The same results are presented in the format of the pruned trees and their outcomes per condition with and without startle are displayed in Figs 1 and 2. High Pain Intensity was associated with higher scores in the body competence subscale of the BCQ (tonic alone), in the fear of severe pain (phasic alone), in the catastrophic thoughts subscale (phasic+ startle) and the confirmation or not of the “conviction of increasing sensitivity” interview-item (“Repeated stimulation causes the stimulated area to become sensitive”) (tonic + startle). High Pain Unpleasantness was associated with higher scores in the body competence subscale (tonic alone) and in the catastrophic thoughts subscale (tonic + startle and phasic + startle) and in the fear of severe pain subscale (phasic alone). Finally, high Pain-Related Suffering was affiliated with lower scores in the coping thoughts subscale of the PRSS questionnaire (tonic alone and tonic + startle), higher total score of all three subscales of the fear of pain FPQ questionnaire (phasic alone) and higher scores of the fear of minor pain subscale (phasic + startle).

**Table 3:** Summary of psychological variables and situational factors predicting intensity, unpleasantness and pain-related suffering in each condition.

	Intensity	Unpleasantness	Pain-related Suffering
**Phasic**	FPQ- Fear of severe pain	FPQ- Fear of severe pain	FPQ- Fear of Pain Total score
**Phasic + Startle**	PRSS-Catastrophizing	PRSS-Catastrophizing	FPQ- Fear of minor pain
**Tonic**	BCQ-Body Competence	BCQ-Body Competence	PRSS- Active Coping
**Tonic + Startle**	Conviction of increasing sensitivity (interview item)	PRSS-Catastrophizing	PRSS- Active Coping

FPQ, Fear of pain questionnaire [34,35]; PRSS, Pain-related self-statements [36–37]; BCQ, Body consciousness scale [41,42].

### Unpruned trees (exploratory model)

Unpruned trees were deliberately computed in the pain-only and not in the startle conditions. These models are exploratory and have no predictive power, demonstrating the detailed decision making progression related to the ratings for each pain dimension. For the sake of brevity, we graphically present the three trees with the simplest distribution, focusing on the phasic condition for I and the tonic condition for U, PS (3). The complete sets of unpruned tree-related data are documented inTables 4 (phasic) and 5 (tonic).

**Table 4:** If-Then rules [59–64] extracted from the unpruned trees for intensity, unpleasantness and pain-related suffering in the phasic condition.

Rules extracted though decision tree in I, U, PS during the PHASIC condition	
**Intensity**	R1: IF low fear of severe pain (FPQs<27.5) THEN: low I (8/8)
	R2: IF high fear of severe pain (FPQs>27.5) and low catastrophic thoughts (PRSSs <1.4) THEN: low I (3/4)
	R3: IF high fear of severe pain (FPQs>27.5) and high catastrophic thoughts (PRSSs >1.4) and “After some time, I did not feel numbness or tingling in the stimulated area” (Item24 = 2) THEN: low I (1/1)
	R4: IF high fear of severe pain (FPQs>27.5) and high catastrophic thoughts (PRSSs >1.4) and “After some time, I felt numbness or tingling in the stimulated area” (Item24≠2) THEN: high I (11/11)
**Unpleasantness**	R1: IF low fear of severe pain (FPQs<27.5) THEN: low U (8/8)
	R2: IF high fear of severe pain (FPQs>27.5) and low private body consciousness (BCQs <12.5) THEN: high U (9/9)
	R3: IF high fear of severe pain (FPQs>27.5) and high private body consciousness (BCQs>12.5) and low body competence (BCQs<8.5) THEN: high U (3/4)
	R4: IF high fear of severe pain (FPQ>27.5) and high private body consciousness (BCQs>12.5) and high body competence (BCQs>8.5) THEN: low U (3/3)
	R1: IF low fear of pain (FPQ<71.5) and “As the intensity goes up, my ability to concentrate decreases” (Item2 = 1) THEN: high PS (1/1)
**Pain-related Suffering**	R2: IF low fear of pain (FPQ<71.5) and “As the intensity goes up, my ability to concentrate does not decrease” (Item2≠1) THEN: low PS (9/9)
	R3: IF high fear of pain (FPQ>71.5) and low fear of medical pain (FPQ<30.5) THEN: high PS (8/8)
	R4: IF high fear of pain (FPQ>71.5) and high fear of medical Pain (FPQ<30.5) and low fear of minor pain (FPQ<23.5) THEN: low PS (2/2)
	R5: IF high fear of pain (FPQ>71.5) and high fear of medical Pain (FPQ<30.5) and high fear of minor pain (FPQ>23.5) THEN: high PS (3/4)

s, Subscale; R, Rule; IF rules THEN [59–64]: Category (low or high) (n° of subjects in this category/total number of subjects under this rule). FPQ, Fear of Pain Questionnaire [34,35]; PRSS, Pain-Related Self-statements Scale [36–37]; BCQ, Body Consciousness Scale: Private, Public and Body Competence [41,42].

**Table 5:** If- then rules [59–64] extracted from the unpruned trees for intensity, unpleasantness and pain-related suffering in the tonic condition.

Rules extracted though decision tree in I, U, PS during the TONIC condition	
**Intensity**	R1: IF low body competence (BCQs<9.5) and low catastrophic thoughts (PRSSs<1.9) THEN: low I (8/8)
	R2: IF low body Competence (BCQs<9.5) and high catastrophic thoughts (PRSSs>1.9) and low public body consciousness (BCQs<12.5) THEN: high I (4/4)
	R3: IF low body competence (BCQs<9.5) and high catastrophic thoughts (PRSSs>1.9) and high public body consciousness (BCQs>12.5) THEN: low I (3/4)
	R4: IF high body competence (BCQs>9.5) and “I cannot take the pain anymore” (Item17 = 1) THEN: low I (1/1)
	R5: IF high body competence (BCQs>9.5) and “I can take the pain” (item17≠1) THEN: high I (7/7)
**Unpleasantness**	R1: IF low body competence (BCQs<8.5) and low coping thoughts (PRSSs<3.1) THEN: high U (3/4)
	R2: IF low body competence (BCQs<8.5) and high coping thoughts (PRSSs>3.1) THEN: low U (9/9)
	R3: IF high body competence (BCQs>8.5) and “As the intensity goes up, my ability to concentrate does not decrease” (Item2 = 2) THEN: low U (1/1)
	R4: IF high body competence (BCQs>8.5) and “As the intensity goes up, my ability to concentrate decreases” (Item2≠2) and low positive affect (PANASs<39.5) THEN: high U (9/9)
	R5: IF high body competence (BCQs>8.5) and “As the intensity goes up, my ability to concentrate decreases” (Item2≠2) and high positive affect (PANASs>39.5) THEN: low U (1/1)
**Pain-related Suffering**	R1: IF low coping thoughts (PRSSs<4.1) and low body competence (BCQs<5) THEN: low PS (3/3)
	R2: IF low Coping thoughts (PRSSs<4.1) and high body competence (BCQs>5) and “I lack control over my body” (Item29 = 1) and low private body consciousness (BCQs<9) THEN: high PS (1/1)
	R3: IF low coping thoughts (PRSSs<4.1) and high body competence (BCQs>5) and “I lack control over my body” (Item29 = 1) and high private body consciousness (BCQs>9) THEN: low PS (2/2)
	R4: IF low coping thoughts (PRSSs<4.1) and high body competence (BCQs>5) and “I do not lack control over my body” (Item29≠1) and low fear of minor pain (FPQs<33) THEN: high PS (11/11)
	R5: IF low coping thoughts (PRSSs<4.1) and high body competence (BCQs>5) and “I do not lack control over my body” (Item29≠1) and high fear of minor pain (FPQs>33) THEN: low PS (1/1)
	R6: IF high coping thoughts (PRSSs>4.1) THEN: low PS (6/6)

s, Subscale; R, Rule; IF rules THEN [59–64]: Category (low or high) (n° of subjects in this category/total number of subjects under this rule). FPQ, Fear of Pain Questionnaire [34,35]; PRSS, Pain-Related Self-statements Scale [36–37]; BCQ, Body Consciousness Scale: Private, Public and Body Competence [41,42]; PANAS, Positive and Negative Affect Schedule [45,46].

**Fig 3:**
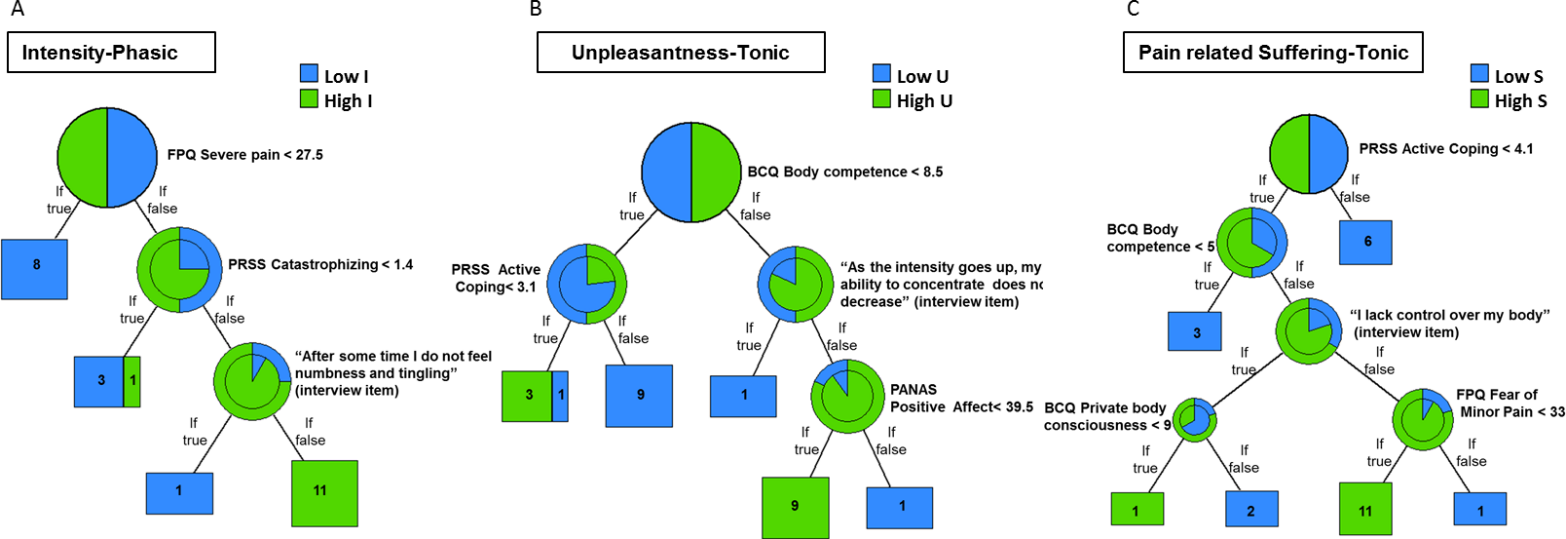
Unpruned trees for intensity ratings in the phasic condition, as well as unpleasantness and pain-related suffering in the tonic condition. Each tree presents the different rules as well as the number of subjects that followed this rule (bottom boxes). The right leaf of the tree represents the amount of subjects who followed the rule (If true), and the left leaf the subjects who did not (If not). In general, the rules have the form: *if* condition1 *and* condition2 *and* condition3 *then* outcome [59–64].

In the first example, the unpruned decision tree for Pain Intensity (Fig 3A, Table 4) revealed that for participants with high fear of pain and high catastrophizing the intensity rated under the less pronounced phasic pain stimulation (generally experienced as less intense) mainly depended on whether the felt pain was accompanied or not by a strange physical sensation. This decision process is exemplified in the next three rules:

1. If the participants had low fear of severe pain (FPQs < 27.5) then they rated low pain intensity (I < 42.73)
  However, the righthand leaf node showed the following split:
2. If the participants had medium-high fear of severe pain (FPQs > 27.5) and high catastrophizing (PRSSs > 1.4), in addition to confirming that “After some time I did feel numbness and tingling” (interview item), then they rated high Pain Intensity (I < 42.73).
3. Yet, if the same process of participants with medium-high fear of severe pain and high catastrophizing was followed by a negation of the final situational item “After some time I did not feel numbness and tingling”, it resulted in the rating of low Pain Intensity (I > 42.73).

Additionally in the second example, the Pain Unpleasantness decision tree (Fig 3B, Table 5), following the more pronounced tonic pain stimulation, revealed that the unpleasantness ratings were mainly associated with the mood state of participants who displayed medium-high bodily competence and altered concentration capacities determined by the lived situation. This decision process is exemplified in the next three rules:

1. If the participants had low scores in bodily competence (BCQs < 8.5) reflecting lower bodily endurance and resistance and reduced coping (PRSS < 3.1) then they rated high Pain Unpleasantness (U > 73.22).
  However, the combination of this psychological predicative construct with situational factors in the right hand leaf node showed a decisional progression whereby:
2. If the participants had medium-high bodily competence (BCQs > 8.5) (high endurance and resistance) followed by maintained concentration “As the intensity goes up, my ability to concentrate does not decrease” (interview item) then they rated low pain unpleasantness (U < 73.22).
3. Yet, when the same progression of participants with medium-high bodily competence followed by reduced concentration “As the intensity goes up, my ability to concentrate decreases” (interview item) and then followed by indications of low to medium positive affect (PANAS Positive < 39.5), high Pain Unpleasantness ratings were rather noted (U > 73.22).

Finally in the third example, the pain-related suffering decision tree (Fig 3C, Table 5) following the more painful tonic stimulation confirmed that scores in low-average coping predicted high suffering ratings. This decision process is exemplified by the following three rules in the left hand leaf node:

1. If the participants had reduced coping abilities (PRSSs < 4.1) but high bodily competence (BCQs > 5) conveying high bodily strength and confirmed “I do not lack control over my body” (interview item) as well as indicated low-medium fear of minor pain, then they had high suffering ratings (PS > 57.07).
  Progressive splits in the same left hand leaf node with different decisional progression however showed that:
2. If the participants had reduced coping (PRSSs < 4.1) but high bodily competence (BCQs > 5), confirming on the contrary “I lack control over my body” and having low body private consciousness (BCQs < 9), then they still rated high suffering (PS > 57.07).
3. At this final stage of the decision making of rule 2, high private body consciousness (BCQs > 9), reflecting enhanced tendency to focus on internal body sensations, resulted however in low suffering ratings (PS < 57.07).

Hence, the suffering ratings mainly depended on the coping thoughts of participants despite having displayed high bodily competence, low fear of pain and alternating between a reported sense of control or lack of control over their body and low versus high body private consciousness.

## Discussion

Our main goal was to explore the factors that may lead individuals to report more pain and pain-related suffering than others. The overall classification accuracy of the predictive trees modeling was 75% for Intensity, 77% for Unpleasantness and 78% for Pain-related Suffering, suggesting that the appraisal of the three pain dimensions was largely determined by psychological constructs. Better prediction was noted for high versus low I, U and PS. An important finding is that the probability of expressing suffering was predominantly associated with fear of pain at different degrees (minor/total) for the less pronounced phasic pain condition and with active coping for the more pronounced tonic pain condition. This outcome suggests that individuals manifesting higher active coping strategies may suffer less [67–69] despite enhanced pain and those who fear pain may suffer even under low pain.

### Variables associated with pain intensity, unpleasantness and suffering

Our second objective consisted in presenting a classification of the factors having the most significant weight on the evaluation of each pain dimension. Results of the predictive pruned model suggest two lines of evidence. For the phasic pain only condition (Table 4), I and U were mainly associated with severe fear of pain (e.g. breaking a leg) while PS was related to the total (representing minor, medical and severe altogether) fear of pain. However, under tonic pain (Table 5), I and U were mainly associated with low-medium body competence (physical strength and resistance) while PS was negatively related to the extent of coping resources. For the tonic pain and startle conditions, the only situational factor from the interview item reporting high ‘conviction of increasing sensitivity’ («Repeated stimulation causes the stimulated area to become sensitive») was associated with enhanced I, describing the process of pain amplification as an increased reaction to continuous and constant stimulation [70]. Another dominant factor was high pain catastrophizing, associated with enhanced I and U for phasic pain and with higher U for tonic pain.

Catastrophizing is one of the most important pain predictors [71], consisting “of extremely negative thoughts about one’s plight” that result in interpreting minor problems as major catastrophes [72]. The functional deficits associated with this psychological trait are accompanied by greater perception of both experimental and clinical pain [73, 72, 74, 75] and are linked to greater emotional distress [76,77]. Specifically, Wade et al. [78] reported catastrophic thoughts to have significant and roughly equal effects on I and U, but also on PS in chronic arthritic knee patients. In our study however, catastrophizing emerged as a marker for I and U rather than PS and only in the pain plus startle conditions. We hypothesize that catastrophizing was rather linked to the psychological impact of the startle probes that were presented in a more unexpected and intermittent fashion than to the continuously applied mechanical pain stimuli. In addition, the very short duration (50ms) of the startle stimuli prevented adequate coping strategies. Under our controlled laboratory environment, the startle paradigm may hence have diverted the attention from pain processing towards unpredictably applied additional stressors, leading to the reporting of U rather than PS.

The second dominant psychological variable was fear of pain, measured prior to being exposed to the experimental protocols. Fear, associated with I, U and principally PS under lower pain-related phasic stimulations could express the expectation of the upcoming pain by conveying “a disposition to respond fearfully to a troubling situation rather than to an actual experience of pain” [79,80]. It is one of the most reliable predictors for chronic pain intensity [71] and is enhanced by cognitive evaluations [19] like reflections on how physically harmful or difficult it may be to endure the pain over time. Accompanied by more pronounced negative emotional and cognitive processing [81,82], its anticipative role [4] of upcoming threat [83,84] may therefore be a signal of PS even under low pain.

Low and high bodily competence was identified as a third characteristic associated with low and high I and U ratings, respectively, under the more painful tonic condition. This may show that the induced pain intensities were judged as greater than initially expected, leading to a disempowering experience and reduced self-management. We found that physiologically oriented construct (bodily competence) or situational item recorded in the interview (high ‘conviction of increasing sensitivity’) were associated with the rating of I for the high pain condition. This suggests that I ratings may primarily depend on bodily strength or endurance while reduced mental strength is more critically involved in the reporting of PS.

Paucity of active coping strategies [36], emerged as the most distinctive attribute associated with PS. While particular measurement of the engagement/avoidance of the pain [85] was not performed, two active behavioral strategies were reported in the coded interviews: ignoring the pain by engaging in the tasks or by diverting attention away from the situation. Participants reported suffering when these efforts resulted in either decreased concentration, reduced self-control (Table 3), enhanced uncertainty or mental strain and exhaustion. This result confirms the significant correlation between reduced psychological-cognitive coping mechanisms and suffering in palliative and lupus patients [86–88] and the identification of coping as a predictor of suffering in advanced cancer patients [89].

Overall, the novelty in our findings consists in providing a meaningful framework for the classification of attributes of pain and related-suffering.

### Modulation by situational factors

Regarding the second objective, our results also emphasize the possible modulation of the importance of the psychological constructs on the decision rating procedure by situational variables. Although decision-making processes underlying pain-related emotional and cognitive responses have rarely been investigated, it was shown that predispositional cognitive functions involve implicit automatic processing rather than deliberate weighing of one’s options [90, 19]. This was highlighted by our pruned model. Yet the flowchart of features denoted in the unpruned model revealed that rating judgments equally depended on changes from situational sources of variation [91]. Several paths manifesting a split in the course of the pain-suffering rating decisions demonstrated the impact of situational factors, collected from the post-experiment interviews.

As illustrated in the pain only conditions, the selected examples of the unpruned trees for I, U and PS respectively show as follows: for the decision tree of Pain Intensity in response to the more moderate phasic stimulation, it is suggested that the prognostic value of fear of pain and catastrophizing may be modified by concomitant sensations like numbness and tingling (situational item)(Table 4 R4). Accordingly, in the Unpleasantness tree model for the more pronounced tonic pain, enhanced Unpleasantness was associated with high bodily competence but equally depended on reduced ability to focus one’s mind (situational item) and the amount of positive mood preceding the pain induction (Table 5 R4, R5). Deviations due to contextual impact were also found in the Pain-related Suffering decision tree following tonic stimulation. Decreased active coping strategies and high bodily competence were associated with suffering but various decision paths demonstrated the impact of situational factors on the respective ratings choice; high suffering was largely maintained despite reported sense of control over one’s body (situational item) and low fear of minor pain (Table 5 R4). Yet, when a reported sense of lack of control over one’s body was followed by either reduced or enhanced body consciousness, PS was changeably classified as either high (Table 5 R2) or low (Table 5 R3) depending on the individual.

Our findings are reminiscent of previous lines of research on person-situation interactions in the framework of psychological assessments [92]. In this study, we have confirmed that psychological constructs are indeed more stable in determining the likelihood for the reporting of pain and pain-related suffering but their weight on implicit rating choices may be altered by contextual factors accordingly modulating the assessment of I-U-PS. The narratives collected during the interviews shed a light on the subjective experience and subsequently on the differences in individual processing of these situational variables. The social context and the situational demand of the investigator were not spontaneously raised by participants during their interviews and thus not included, even though they potentially influenced [93] the pain and suffering report.

### Limitations of the study

As a limitation, the results of the present experimental study may only be valid for healthy volunteers, acknowledging that what we most seek to elucidate in the long term is ongoing clinical pain that is commonly accompanied by comorbid mental health disorders. Also, due to the small sample size of the present exploratory and descriptive study, the results need to be replicated with larger groups of participants, even though the experiment was repeated twice for each method (with and without concomitant startle probes). In spite of the small number of participants and the large amount of assessed variables, the decision tree technique selects the "best" attributes as a way to extract predictive features. As a method combining qualitative and quantitative measures, it therefore has the advantage of documenting the direct impact of psychobiological factors on the entire range of I-U-PS scores (inconsistently classified to date [94, 12, 19, 26]; for review see [95]). Last is the bias limitation of a latent component revealed since suggested, estimating that inquiring about suffering might necessarily entail reports of suffering and that the differentiation between unpleasantness and suffering is the outcome of the questioning. Yet given our findings [2,3] not all participants reported suffering in all methods at all times while clearly dissociating suffering from unpleasantness. It may be noted here that the quantitative ratings of I, U, PS were compared with their interpretation provided by the qualitative data of the interviews, explaining what they meant, with items clearly attesting to a conceptual difference between all pain components [95].

## Conclusions

The decision tree technique used here led to the development of a highly accurate classification of attributes of Pain Intensity, Unpleasantness and Suffering response behaviour. We have provided evidence that situational factors need to be considered in addition to the commonly examined predictors related to psychological characteristics. The originality of our approach also lies in a classification of distinctive factors most strongly determining the evaluation of each pain dimension and stresses the relevance of evaluating pain-related suffering even in healthy volunteers, provided that the experimentally induced pain mimics clinical pain. Additional potential predictors should be addressed. Based on our experimental results regarding physiological suffering measurements (e.g. startle), further studies should include the utility of these objective measures as predictors of suffering to back up the self-report evaluations. Data from this study on the effect of fear of pain (equally found in Brunner et al. [3]) and of active cognitive coping may represent an important step in the prognosis of individual suffering. Cautiously considering that while “pain is inevitable, suffering is optional” [96], it may be suggested that this outcome highlights the importance of improving cognitive coping strategies in clinical settings.

## Supporting information

S1 Table Participants’ questionnaire data and pain thresholds. Mean (M) ± standard deviation (SD) and range, in parenthesis, are displayed for all participants.
(DOCX)

S2 Table Participants’ questionnaire data examining the mood state of the participant before the main experiment. Mean ± standard deviation and range (min-max), are displayed for all participants.
(DOCX)

S1 File Demographic, psychophysical and questionnaire-related data sets. (XLS)
