# Supplementary material for: Psychological, cognitive factors and contextual influences in pain and pain-related suffering as revealed by a combined qualitative and quantitative assessment approach
Source: PLoS One. 2018 Jul 31;13(7):e0199814. doi: 10.1371/journal.pone.0199814 (PMC6067693; doi:10.1371/journal.pone.0199814)
Supplement: S1 Table — Mean (M) ± standard deviation (SD) and range, in parenthesis, are displayed for all participants. (DOCX) [file pone.0199814.s001.docx]

**S1 Table.** Participants’ questionnaire data and pain thresholds. Mean (M) ± standard deviation (SD) and range, in parenthesis, are displayed for all participants.

| **Measure** | **Total sample**  **M ± SD (range)**  **n=24** |
| --- | --- |
| Tonic method: Pain Threshold (in Newtons) | 1.89 ± 0.73 (1-3.90) |
| Tonic method: Pain Tolerance (in Newtons) | 3.81 ± 0.92 (2.20-5.20) |
| Phasic method: Pain Threshold (in g^.^m^.^s^-1^) | 1.57 ± 0.34 (1.15-2.70) |
| Phasic method: Pain Tolerance (in g^.^m^.^s^-1^) | 2.24 ± 0.39 (1.58-2.70) |
| FPQ-Minor Pain | 18.17 ±7.57 (20-36) |
| FPQ-Medical Pain | 25.21 ± 7.91 (10-39) |
| FPQ-Severe Pain | 31.92 ± 9.8 (13-45) |
| PRSS-catastrophizing | 2 ± 0.75 (1-3.33) |
| PRSS-active coping | 3.58 ± 0.58 (2.44-4.44) |
| BCQ - Private body consciousness | 11.65 ± 3.88 (4-20) |
| BCQ - Public body consciousness | 13.43 ± 4.79 (5-24) |
| BCQ - Body competence | 8.35 ± 2.95 (1-15) |
