# Supplementary material for: Psychological, cognitive factors and contextual influences in pain and pain-related suffering as revealed by a combined qualitative and quantitative assessment approach
Source: PLoS One. 2018 Jul 31;13(7):e0199814. doi: 10.1371/journal.pone.0199814 (PMC6067693; doi:10.1371/journal.pone.0199814)
Supplement: S2 Table — Mean ± standard deviation and range (min-max), are displayed for all participants. (DOCX) [file pone.0199814.s002.docx]

**S2 Table.** Participants’ questionnaire data examining the mood state of the participant before the main experiment. Mean ± standard deviation and range (min-max), are displayed for all participants.

|  | Valence | Arousal | Dominance | Negative Affect | Positive Affect | How worried are you that something serious might happen?  (min 1- max 5) | How afraid are you of the following pain induction?  (min 1- max 5) |
| --- | --- | --- | --- | --- | --- | --- | --- |
| Tonic | 4,39 ± 1,56  1-8 | 3,83 ± 1,77  1-7 | 4,61 ± 0,94  3-7 | 14,43 ± 4,03  10-24 | 28,43 ± 7,71  10-42 | 1,52 ± 0,85  1-4 | 2,04 ± 0,82 |
| Tonic + Startle | 4,04 ± 1,40  2-7 | 3,29 ± 1,78  1-8 | 4,83 ± 1,30  3-8 | 15,08 ± 6,29  10-37 | 30,25 ± 8,44  13-43 | 1,30 ± 0,76  1-4 | 1,57 ± 0,51  1-2 |
| Phasic | 4,25 ± 1,51  1-7 | 3,25 ± 1,70  1-7 | 4,63 ± 0,88  3-6 | 14,96 ± 6,15  10-38 | 30,13 ± 7,81  11-43 | 1,33 ± 0,76  1-4 | 1,83 ± 0,76  1-3 |
| Phasic + Startle | 4,04 ± 1,23  2-6 | 3,54 ± 2,00  1-9 | 5,00 ± 0,88  3-7 | 14,58 ± 4,41  10-25 | 29,33 ± 7,91  17-45 | 1,42 ± 0,78  1-4 | 1,67 ± 0,87  1-4 |
